# Supplementary material for: Two-sample survival tests based on control arm summary statistics
Source: PLoS One. 2024 Jun 14;19(6):e0305434. doi: 10.1371/journal.pone.0305434 (PMC11178201; doi:10.1371/journal.pone.0305434)
Supplement: S1 Appendix — Mathematical statements and corresponding proofs. (PDF) [file pone.0305434.s001.pdf]

One-sample log-rank tests with consideration of reference curve sampling variability: S1 Appendix

Jannik Feld[1], Moritz Fabian Danzer[1], Andreas Faldum[1], Rene Schmidt[1],

1 Institute of Biostatistics and Clinical Research, University of Münster, 48149 Münster, Germany

* Jannik.Feld@ukmuenster.de

Appendix A: Simulation results for different effect sizes

Table 1. Comparison of empirical type I and II errors of the new procedure and two-sample log-rank test with varying follow-up length

		$f_B = 2$					$f_B = 3$					$f_B = 4$				
κ	n_A	n_B	α_{TS}	α_π	$1-\beta_{TS}$	$1-\beta_\pi$	n_B	α_{TS}	α_π	$1-\beta_{TS}$	$1-\beta_\pi$	n_B	α_{TS}	α_π	$1-\beta_{TS}$	$1-\beta_\pi$
0.10	400	677	0.045	0.044	0.715	0.710	651	0.050	0.050	0.716	0.715	633	0.051	0.054	0.716	0.708
	800	438	0.049	0.049	0.754	0.762	427	0.051	0.050	0.751	0.761	418	0.051	0.052	0.749	0.754
0.25	400	529	0.048	0.046	0.727	0.724	489	0.051	0.051	0.713	0.716	462	0.049	0.054	0.710	0.700
	800	369	0.046	0.045	0.751	0.760	349	0.051	0.052	0.754	0.765	334	0.048	0.051	0.756	0.757
0.50	400	382	0.046	0.043	0.733	0.735	340	0.051	0.051	0.729	0.738	314	0.046	0.053	0.725	0.709
	800	289	0.050	0.049	0.760	0.770	264	0.051	0.051	0.753	0.768	248	0.049	0.051	0.745	0.742
1.00	400	253	0.052	0.050	0.736	0.743	227	0.050	0.050	0.730	0.742	214	0.053	0.052	0.718	0.714
	800	208	0.046	0.045	0.752	0.766	190	0.049	0.049	0.752	0.766	181	0.053	0.052	0.745	0.749
1.50	200	326	0.049	0.047	0.693	0.681	301	0.053	0.048	0.676	0.657	295	0.048	0.043	0.675	0.640
	400	214	0.053	0.051	0.723	0.730	202	0.049	0.046	0.727	0.727	200	0.053	0.050	0.724	0.717
	800	180	0.053	0.052	0.744	0.757	172	0.051	0.048	0.740	0.752	170	0.050	0.047	0.739	0.745
2.00	200	302	0.056	0.050	0.686	0.670	295	0.053	0.048	0.683	0.653	294	0.050	0.045	0.677	0.647
	400	203	0.045	0.042	0.729	0.731	199	0.051	0.049	0.722	0.717	199	0.050	0.046	0.722	0.720
	800	173	0.050	0.049	0.743	0.755	170	0.056	0.053	0.740	0.749	170	0.048	0.044	0.735	0.744
5.00	200	294	0.049	0.044	0.685	0.653	294	0.050	0.046	0.679	0.644	294	0.051	0.046	0.679	0.646
	400	199	0.049	0.045	0.722	0.719	199	0.048	0.046	0.719	0.716	199	0.049	0.045	0.721	0.715
	800	170	0.054	0.050	0.746	0.755	170	0.047	0.045	0.741	0.751	170	0.047	0.044	0.741	0.751

Empirical type I error rates (α_π and α_{TS}) and powers ($1-\beta_\pi$ and $1-\beta_{TS}$) for the new test and for the classical two-sample log-rank test, respectively, under proportional hazards alternatives for Weibull distributed survival times with shape parameter κ and 1-year survival rate $S_1 = 0.5$ in the control arm. Theoretical two-sided significance level: 5%. Underlying sample size of the historical group n_A was predefined whereas the sample size of the intervention group n_B was calculated to achieve a theoretical power of 80% under the planning alternative $H_1 : \Lambda_B = 4/5 \cdot \Lambda_A$ for the new test statistic using the sample-size methodology presented in the previous section. No censoring despite administrative censoring after $f_A = 3$ and f_B years in the historical and intervention group respectively.

Table 2. Comparison of empirical type I and II errors of the new procedure and two-sample log-rank test with varying censoring mechanism

		$\lambda_{C_B} = 0.15$					$\lambda_{C_B} = 0.20$					$\lambda_{C_B} = 0.25$				
κ	n_A	n_B	α_{TS}	α_π	$1-\beta_{TS}$	$1-\beta_\pi$	n_B	α_{TS}	α_π	$1-\beta_{TS}$	$1-\beta_\pi$	n_B	α_{TS}	α_π	$1-\beta_{TS}$	$1-\beta_\pi$
0.10	400	663	0.054	0.058	0.707	0.711	667	0.048	0.050	0.697	0.704	671	0.050	0.053	0.702	0.706
	800	432	0.047	0.048	0.749	0.760	433	0.049	0.050	0.746	0.760	435	0.048	0.050	0.751	0.763
0.25	400	507	0.050	0.057	0.705	0.722	513	0.049	0.055	0.696	0.713	519	0.052	0.058	0.694	0.710
	800	358	0.052	0.057	0.740	0.759	361	0.046	0.050	0.746	0.767	364	0.049	0.053	0.744	0.761
0.50	400	358	0.048	0.059	0.704	0.733	364	0.052	0.061	0.701	0.728	370	0.054	0.062	0.700	0.723
	800	275	0.051	0.056	0.735	0.762	279	0.050	0.054	0.738	0.765	282	0.048	0.053	0.736	0.760
1.00	400	240	0.048	0.059	0.685	0.715	245	0.051	0.059	0.681	0.710	249	0.053	0.060	0.688	0.716
	800	199	0.048	0.053	0.735	0.764	202	0.048	0.053	0.739	0.767	206	0.050	0.053	0.729	0.756
1.50	200	325	0.053	0.062	0.627	0.623	333	0.047	0.056	0.628	0.621	342	0.050	0.059	0.628	0.623
	400	213	0.051	0.055	0.686	0.697	217	0.050	0.054	0.686	0.695	221	0.050	0.053	0.697	0.706
	800	180	0.050	0.052	0.720	0.736	183	0.048	0.051	0.728	0.746	185	0.056	0.058	0.726	0.741
2.00	200	316	0.052	0.060	0.629	0.620	323	0.052	0.058	0.632	0.616	331	0.054	0.058	0.636	0.620
	400	209	0.050	0.053	0.694	0.701	212	0.052	0.055	0.698	0.704	216	0.053	0.056	0.686	0.690
	800	177	0.054	0.055	0.724	0.740	180	0.053	0.054	0.742	0.755	182	0.053	0.055	0.723	0.737
5.00	200	312	0.049	0.055	0.637	0.628	319	0.050	0.056	0.639	0.629	325	0.051	0.056	0.632	0.618
	400	207	0.051	0.053	0.694	0.702	210	0.052	0.055	0.693	0.699	213	0.049	0.052	0.699	0.703
	800	176	0.048	0.049	0.735	0.748	178	0.050	0.052	0.723	0.739	180	0.055	0.056	0.734	0.748

Empirical type I error rates (α_π and α_{TS}) and powers ($1-\beta_\pi$ and $1-\beta_{TS}$) for the new test and for the classical two-sample log-rank test, respectively, under proportional hazards alternatives for Weibull distributed survival times with shape parameter κ and 1-year survival rate $S_1 = 0.5$ in the control arm. Theoretical two-sided significance level: 5%. Underlying sample size of the historical group n_A was predefined whereas the sample size of the intervention group n_B was calculated to achieve a theoretical power of 80% under the planning alternative $H_1 : \Lambda_B = 4/5 \cdot \Lambda_A$ for the new test statistic using the sample-size methodology presented in the previous section. Beside administrative censoring after $f_A = f_B = 3$ years in the historical and intervention group, we also introduced random Exponential distributed censoring with rates $\lambda_{C_A} = 0.20$ and λ_{C_B} in the historical and intervention group respectively.

Table 3. Comparison of empirical type I and II errors of the new procedure and two-sample log-rank test with varying follow-up length

		$f_B = 2$					$f_B = 3$					$f_B = 4$				
κ	n_A	n_B	α_{TS}	α_π	$1-\beta_{TS}$	$1-\beta_\pi$	n_B	α_{TS}	α_π	$1-\beta_{TS}$	$1-\beta_\pi$	n_B	α_{TS}	α_π	$1-\beta_{TS}$	$1-\beta_\pi$
0.10	200	48	0.053	0.050	0.771	0.814	47	0.050	0.048	0.774	0.816	46	0.048	0.049	0.767	0.807
	400	44	0.054	0.052	0.782	0.829	43	0.050	0.048	0.779	0.830	42	0.049	0.048	0.774	0.822
	800	42	0.050	0.050	0.784	0.840	41	0.052	0.051	0.774	0.834	41	0.051	0.051	0.775	0.832
0.25	200	41	0.049	0.046	0.774	0.816	38	0.054	0.052	0.760	0.810	37	0.051	0.050	0.764	0.807
	400	38	0.052	0.052	0.779	0.832	36	0.050	0.047	0.772	0.825	34	0.050	0.052	0.762	0.812
	800	36	0.050	0.050	0.766	0.827	35	0.051	0.049	0.782	0.836	33	0.051	0.047	0.774	0.829
0.50	200	31	0.048	0.046	0.755	0.802	28	0.053	0.050	0.755	0.804	26	0.056	0.050	0.729	0.778
	400	30	0.056	0.054	0.760	0.817	27	0.056	0.054	0.758	0.817	25	0.054	0.049	0.746	0.799
	800	29	0.052	0.048	0.762	0.819	26	0.052	0.049	0.762	0.820	25	0.049	0.048	0.766	0.817
1.00	200	21	0.058	0.048	0.740	0.792	18	0.056	0.050	0.714	0.767	17	0.056	0.045	0.702	0.750
	400	20	0.063	0.056	0.739	0.801	18	0.058	0.049	0.734	0.787	16	0.057	0.047	0.698	0.754
	800	20	0.057	0.053	0.741	0.798	18	0.059	0.050	0.743	0.800	16	0.056	0.046	0.708	0.767
1.50	200	17	0.056	0.046	0.711	0.761	15	0.061	0.045	0.682	0.728	15	0.059	0.043	0.690	0.718
	400	16	0.059	0.047	0.703	0.762	15	0.059	0.047	0.715	0.770	14	0.060	0.045	0.678	0.725
	800	16	0.058	0.050	0.717	0.775	14	0.057	0.044	0.679	0.738	14	0.059	0.045	0.694	0.747
2.00	200	15	0.061	0.049	0.691	0.735	14	0.060	0.045	0.669	0.704	14	0.063	0.047	0.666	0.699
	400	15	0.061	0.045	0.701	0.749	14	0.062	0.048	0.681	0.729	14	0.061	0.046	0.690	0.734
	800	14	0.062	0.050	0.689	0.745	14	0.059	0.045	0.697	0.748	14	0.058	0.046	0.693	0.747
5.00	200	14	0.061	0.046	0.664	0.697	14	0.063	0.046	0.664	0.697	14	0.058	0.044	0.670	0.702
	400	14	0.060	0.045	0.684	0.729	14	0.057	0.044	0.681	0.723	14	0.059	0.042	0.686	0.730
	800	14	0.061	0.046	0.692	0.744	14	0.060	0.043	0.700	0.749	14	0.064	0.046	0.699	0.745

Empirical type I error rates (α_π and α_{TS}) and powers ($1-\beta_\pi$ and $1-\beta_{TS}$) for the new test and for the classical two-sample log-rank test, respectively, under proportional hazards alternatives for Weibull distributed survival times with shape parameter κ and 1-year survival rate $S_1 = 0.5$ in the control arm. Theoretical two-sided significance level: 5%. Underlying sample size of the historical group n_A was predefined whereas the sample size of the intervention group n_B was calculated to achieve a theoretical power of 80% under the planning alternative $H_1 : \Lambda_B = 1/2 \cdot \Lambda_A$ for the new test statistic using the sample-size methodology presented in the previous section. No censoring despite administrative censoring after $f_A = 3$ and f_B years in the historical and intervention group respectively.

Table 4. Comparison of empirical type I and II errors of the new procedure and two-sample log-rank test with varying censoring mechanism

		$\lambda_{C_B} = 0.15$					$\lambda_{C_B} = 0.20$					$\lambda_{C_B} = 0.25$				
κ	n_A	n_B	α_{TS}	α_π	$1-\beta_{TS}$	$1-\beta_\pi$	n_B	α_{TS}	α_π	$1-\beta_{TS}$	$1-\beta_\pi$	n_B	α_{TS}	α_π	$1-\beta_{TS}$	$1-\beta_\pi$
0.10	200	47	0.050	0.052	0.772	0.813	48	0.050	0.048	0.773	0.817	48	0.048	0.050	0.777	0.818
	400	44	0.051	0.048	0.776	0.824	44	0.054	0.053	0.776	0.831	44	0.051	0.051	0.782	0.828
	800	42	0.052	0.051	0.781	0.836	42	0.054	0.052	0.781	0.833	42	0.049	0.046	0.772	0.829
0.25	200	39	0.053	0.056	0.756	0.808	40	0.050	0.051	0.763	0.813	40	0.049	0.052	0.764	0.812
	400	37	0.053	0.050	0.780	0.836	37	0.051	0.050	0.770	0.822	37	0.057	0.056	0.763	0.818
	800	35	0.052	0.053	0.770	0.828	36	0.057	0.056	0.775	0.833	36	0.051	0.048	0.771	0.826
0.50	200	30	0.057	0.056	0.746	0.800	30	0.050	0.049	0.745	0.799	30	0.053	0.051	0.736	0.792
	400	28	0.055	0.052	0.757	0.814	28	0.052	0.052	0.750	0.812	29	0.056	0.052	0.755	0.811
	800	27	0.057	0.053	0.750	0.811	28	0.051	0.048	0.759	0.818	28	0.051	0.050	0.763	0.817
1.00	200	19	0.061	0.053	0.695	0.755	20	0.058	0.051	0.713	0.768	20	0.058	0.051	0.711	0.765
	400	19	0.056	0.050	0.728	0.789	19	0.059	0.049	0.709	0.771	19	0.059	0.052	0.715	0.779
	800	18	0.057	0.048	0.723	0.783	19	0.058	0.049	0.731	0.793	19	0.059	0.051	0.728	0.786
1.50	200	16	0.058	0.044	0.682	0.724	16	0.056	0.044	0.678	0.716	16	0.059	0.043	0.665	0.707
	400	15	0.056	0.041	0.678	0.728	16	0.061	0.049	0.700	0.752	16	0.058	0.045	0.695	0.746
	800	15	0.062	0.050	0.692	0.747	15	0.060	0.044	0.681	0.735	16	0.058	0.046	0.701	0.758
2.00	200	15	0.060	0.046	0.669	0.698	15	0.057	0.041	0.654	0.684	15	0.058	0.042	0.645	0.678
	400	15	0.059	0.044	0.693	0.737	15	0.060	0.041	0.682	0.725	15	0.057	0.044	0.677	0.719
	800	14	0.058	0.045	0.677	0.729	15	0.059	0.044	0.695	0.746	15	0.057	0.045	0.691	0.741
5.00	200	15	0.061	0.045	0.676	0.703	15	0.062	0.049	0.670	0.701	15	0.058	0.043	0.651	0.681
	400	14	0.058	0.044	0.663	0.706	15	0.057	0.044	0.697	0.737	15	0.058	0.042	0.691	0.732
	800	14	0.058	0.044	0.678	0.730	14	0.058	0.043	0.678	0.729	15	0.062	0.046	0.692	0.742

Empirical type I error rates (α_π and α_{TS}) and powers ($1-\beta_\pi$ and $1-\beta_{TS}$) for the new test and for the classical two-sample log-rank test, respectively, under proportional hazards alternatives for Weibull distributed survival times with shape parameter κ and 1-year survival rate $S_1 = 0.5$ in the control arm. Theoretical two-sided significance level: 5%. Underlying sample size of the historical group n_A was predefined whereas the sample size of the intervention group n_B was calculated to achieve a theoretical power of 80% under the planning alternative $H_1 : \Lambda_B = 1/2 \cdot \Lambda_A$ for the new test statistic using the sample-size methodology presented in the previous section. Beside administrative censoring after $f_A = f_B = 3$ years in the historical and intervention group, we also introduced random Exponential distributed censoring with rates $\lambda_{C_A} = 0.20$ and λ_{C_B} in the historical and intervention group respectively.
