# Supplementary material for: Two-sample survival tests based on control arm summary statistics
Source: PLoS One. 2024 Jun 14;19(6):e0305434. doi: 10.1371/journal.pone.0305434 (PMC11178201; doi:10.1371/journal.pone.0305434)
Supplement: Attachment — Submitted filename: Review-PONE-D-23-29714.pdf [file pone.0305434.s007.pdf]

Review of PLOS ONE manuscript PONE-D-23-29714

„On historically controlled survival trials“

Overall, this is an interesting, well-written and relevant manuscript.

General comments

1. I think the title of the manuscript should be more specific. It should include „log-rank test“.
2. To demonstrate practical relevance, I suggest that the manuscript might benefit from a brief additional paragraph on a real world example,showing how the conclusion (the p-value) from a specific phase II trial would change, had the new test been applied.
3. The description of the censoring scheme assumed in the sample size calculation and the simulation study is unclear (see specific comments below). Is it recruitment period plus fixed follow-up or recruitment period plus minimum follow-up until end of trial? Both is found in practice trials with survival endpoints.
4. While it is true that the new test can be implemented when only the historic sample size and the Nelson-Aalen (or 1 minus Kaplan-Meier at maximum follow-up) of the control group is known, I wonder how relevant and accessible the assumption of identical follow-up schemes is in practice. This aspect should receive some more room in the discussion.

Specific comments – being a non-native speaker myself, I may be wrong with the corrections that I propose concerning the English language.

- Abstract, It thus applies in settings, when -> It thus applies in settings when (omit German comma)
- L. 17 This may be for a variety reasons -> This may occur for a variety of reasons
- L. 20 an survival distribution estimator -> a survival distribution estimator
- L. 27 Please rearrange references in numerical order of citation
- L. 30 an estimator of historical survival curves -> Should this read „of the historical survival curve“ (singular, there is only one control group)?
- L. 51/52: Please add „for i in N_x“, as in the sum in line 61.
- L. 53 In addition, and also as usual, one would assume that the data are i.i.d. samples from groups A and B. You require identical distributions in L. 69, but not independence.
- Equn (2): I suppose N_i(s) should read N_x,i(s)?
- L. 64 Please add „for s greater-equal 0“
- L. 65 ff. The „d“ in integrals would preferrably not be set in italics, for clarity.
- L.79, please introduce s_max
- L. 95.5 (equation): Please introduce X_B,i
- M_0-hat is defined twice, in L.101 and equation (5)
- L. 115, stochastic processes is -> stochastic process is
- L. 116, it was shown, that -> it was shown that (omit German comma)
- L. 121.5, leads to a under-estimation -> leads to an under-estimation
- L. 128 and ff, Notice, that -> Notice that (omit German comma)
- L. 134 or equivalent the Kaplan Meier estimator -> or equivalently the Kaplan-Meier estimator
- L. 138.5 You need to introduce Λ_x, probably as Λ_x(s_max)
- L. 140, please introduce a_B, f_B, r_B. You might use terms like recruitment period and fixed-time or additional follow-up.
- L.151,it’s not quite clear where you assume the plateau, in particular since f_B has not been introduced - I don’t understand ‚at the edge of the observation window‘ – does that mean AT s_max? If the plateau occurs during the interval between a_B and a_B+f_B, then (ii) would equal S(a_B) not S(f_B). Please clarify. Also, I’m not sure whether „No censoring despite administrative censoring“ means what I would call „No censoring except for administrative censoring“
- L. 154 declines short after the procedure -> declines shortly after the procedure
- L. 201, independent exponential distributed censoring -> independent exponentially distributed censoring

- L. 208, please indicate also the one-year survival rate in the intervention group in this scenario (S_T_B(1)).
- L 229, scenarios with particular small sample sizes -> scenarios with particularly small sample sizes
- L 270, we advice to use our new test -> we advise to use our new test
- Legend to table 1, „Underlying sample size of the historical group n_A and the intervention n_B calculated to achieve a theoretical power“: I think n_A should be viewed as given historically, while n_B is calculated to achieve a theoretical power. Please rephrase.
