# Supplementary material for: Two-sample survival tests based on control arm summary statistics
Source: PLoS One. 2024 Jun 14;19(6):e0305434. doi: 10.1371/journal.pone.0305434 (PMC11178201; doi:10.1371/journal.pone.0305434)
Supplement: Attachment — Submitted filename: Response to Reviewers.docx [file pone.0305434.s008.docx]

Answer to editor and reviewer comments

concerning the manuscript ‘On historically controlled survival trials’

now titled ‘Designing one-sample log-rank tests in the absence of historic individual patient data’

(PONE-D-23-29714)

We revised the manuscript, following the very detailed comments of the reviewers. We aimed to fully implement and consider their very helpful suggestions, as will be detailed below in our answers to the reviewer comments. In particular, we reduced the overlap to our previous paper considerably, made the use-case of our new methodology more clear and discussed the differences to existing log-rank methodology in more detail within an example with real world data. All changes made are highlighted in red color in the ”track changes” version of the manuscript.

**1 Comments of the editor**

(1) The title should be changed to describe the content of the paper (see suggestions of the reviewers).

Answer:
Thank you for the comment, as the reviewers suggested, we changed the manuscript title.

(2) The differences of the new (corrected one-sample) test and the usual (two-sample) log-rank test should be worked out more thoroughly. Which information from the historical reference curve cannot be extracted to apply the usual (two-sample) log-rank test?

Answer:
Thank you for the comment. We added an example section where we went into detail where the differences are.

(3) The repetitions from earlier papers should be reduced.

Answer:
Thank you for the comment. In the previous version we tried to be as consistent with the previous paper on which our new results are based on. Now we reduced the notation, motivation and testing problem to avoid repetition.

(4) The information that sample-size formulas are propsed and a simulation study was performed should be added to the Abstract

Answer:
As suggested, we added this info to the abstract.

(5) An example should be added illustrating the application of the new method compared to the usual one-sample log-rank and two-sample log-rank tests.

Answer:
Thank you for the suggestion. We added an example with real world data.

(6) The new test called corrected one-sample log-rank test can also be viewed as new two-sample test for the case that complete historical survival data on patient level are not available. However, with this interpretation another important difference between the two data situations is that the classical two-sample log-rank test is usually applied in RCTs, whereas the new test is applied in a non-randomised data situation. The increased uncertainty due to missing randomisation in applications of the new test should be discussed at the end of the paper.

Answer:
Thank you for the remark. We added this point to our discussion.

4. We are unable to open your Supporting Information file Simulation_Function.R, Sample_size_Pi.R, Simulation_Different_Censoring.R and Simulation_Different_FollowUp.R. Please kindly revise as necessary and re-upload.

Answer:
We now uploaded the files as .txt – files. To execute them you will need to open them in an R capable IDE - for example in R-Studio.

**Reviewer #1:** 


1. I think the title of the manuscript should be more specific. It should include „log-rank test“.

Answer:
Thank you for the comment. As suggested we changed the title of the manuscript.

2. To demonstrate practical relevance, I suggest that the manuscript might benefit from a brief additional paragraph on a real world example, showing how the conclusion (the p-value) from a specific phase II trial would change, had the new test been applied.

Answer:
Thank you. We added a new section covering an example with real world data on which we go in detail what the different analysis strategies are and what the p-values would be.

3. The description of the censoring scheme assumed in the sample size calculation and the simulation study is unclear (see specific comments below). Is it recruitment period plus fixed follow-up or recruitment period plus minimum follow-up until end of trial? Both is found in practice trials with survival endpoints.

Answer:
We thank you for the comment. The confusion was probably due to the fact that we forgot to define the variables a and f. It is meant as recruitment period length a plus minimumF follow up period length f resulting in a trial length of a+f.

4. While it is true that the new test can be implemented when only the historic sample size and the Nelson-Aalen (or 1 minus Kaplan-Meier at maximum follow-up) of the control group is known, I wonder how relevant and accessible the assumption of identical follow-up schemes is in practice. This aspect should receive some more room in the discussion.

Answer:
We think that the assumption of similar recruitment and follow-up schemes may be justified since a comparison is only meaningful if the two groups (experimental and historic) are drawn in theory from the same underlying basic population, which in turn influences the censoring and recruitment scheme. Administrative censoring and recruitment period length should of course be chosen to match the historical example as plannable parameters. In addition, we have shown in the simulations that our methodology can maintain type I error rate control quite strictly even under slight misspecifications.

Specific comments – being a non-native speaker myself, I may be wrong with the corrections that I propose concerning the English language.

• Abstract, It thus applies in settings, when -> It thus applies in settings when (omit German comma)
• L. 17 This may be for a variety reasons -> This may occur for a variety of reasons
• L. 20 an survival distribution estimator -> a survival distribution estimator
• L. 27 Please rearrange references in numerical order of citation
• L. 30 an estimator of historical survival curves -> Should this read „of the historical survival curve“ (singular, there is only one control group)?
• L. 51/52: Please add „for i in N_x“, as in the sum in line 61.
• L. 53 In addition, and also as usual, one would assume that the data are i.i.d. samples from groups A and B. You require identical distributions in L. 69, but not independence.
• Equn (2): I suppose N_i(s) should read N_x,i(s)?
• L. 64 Please add „for s greater-equal 0“
• L. 65 ff. The „d“ in integrals would preferrably not be set in italics, for clarity.
• L.79, please introduce s_max
• L. 95.5 (equation): Please introduce X_B,i
• M_0-hat is defined twice, in L.101 and equation (5)
• L. 115, stochastic processes is -> stochastic process is
• L. 116, it was shown, that -> it was shown that (omit German comma)
• L. 121.5, leads to a under-estimation -> leads to an under-estimation
• L. 128 and ff, Notice, that -> Notice that (omit German comma)
• L. 134 or equivalent the Kaplan Meier estimator -> or equivalently the Kaplan-Meier estimator
• L. 138.5 You need to introduce Λ_x, probably as Λ_x(s_max)
• L. 140, please introduce a_B, f_B, r_B. You might use terms like recruitment period and fixed-time or additional follow-up.
• L.151,it’s not quite clear where you assume the plateau, in particular since f_B has not been introduced - I don’t understand ‚at the edge of the observation window‘ – does that mean AT s_max? If the plateau occurs during the interval between a_B and a_B+f_B, then (ii) would equal S(a_B) not S(f_B). Please clarify. Also, I’m not sure whether „No censoring despite administrative censoring“ means what I would call „No censoring except for administrative censoring“
• L. 154 declines short after the procedure -> declines shortly after the procedure
• L. 201, independent exponential distributed censoring -> independent exponentially distributed censoring
• L. 208, please indicate also the one-year survival rate in the intervention group in this scenario (S_T_B(1)).
• L 229, scenarios with particular small sample sizes -> scenarios with particularly small sample sizes
• L 270, we advice to use our new test -> we advise to use our new test
• Legend to table 1, „Underlying sample size of the historical group n_A and the intervention n_B calculated to achieve a theoretical power“: I think n_A should be viewed as given historically, while n_B is calculated to achieve a theoretical power. Please rephrase.

Answer:
Thank you for the many remarks. We edited the text passages in question.

**Reviewer #2:** 
1. It should be made clearer what are the differences from the previous paper (Ref. 12). As far as I can tell, the first sections (until but excluding the section on Sample-size calculation) are repetitions of content from Ref. 6. Also, it should be made more explicit in the section on Sample-size calculation which results are known from the literature (e.g., Ref. 4) an which results are new. Having said this, I am wondering whether the new content is sufficient for justification of a publication in PLOS ONE. But this is not for me to decide. Either way, I believe the paper could benefit from a more specific title (and abstract) which highlights the new contributions (and an abstract which distinguishes a bit better from previous works). One candidate title could be: "Sample size planning for the one-sample log-rank test with a historical control."

Answer:
Thank you for your comment. In the previous draft we wanted to stay notation-wise consistent with our previous paper. In doing so, we adopted a lot of notation that does not necessarily have to be defined in the new paper. We reduced the notation, motivation and testing procedure sections considerably and think that the paper reads more straight forward now. Thank you for this remark.

Regarding the sample-size section: We added further information on difference of the existing sample size methodology and our new one. However the main focus of the new paper was not the sample-size formula but the description and benchmark of the new test methodology which can be used in situations where the classical one-sample and two-sample log-rank tests are not applicable.

2. What is (/are) the real difference(s) between the proposed adjusted "one-sample" test and the two-sample test? (Also the simulation results are very similar for both kinds of tests.) To my understanding, all the relevant data from the historical reference sample can be extracted from the historical reference curve, so it seems like a classical two-sample problem to me. What is more, the two-sample test is known to have a test statistic which is approximately N(0,1)-distributed under the null hypothesis. To my understanding, the authors suggested a one-sample variant which uses an alternative variance estimator to account for the factual two-sample problem. Now, if the proposed test statistic is also approximately N(0,1)-distributed, does not imply that the proposed test approximates the classical two-sample test? It should be made clearer what are the actual differences between both tests. For example, will the difference of the variance estimators decrease to 0 rapidly? I think a good understanding of this is of the essence to underline the relevance of this paper.

Answer:
The TSLR can only be used if the patient level data is at hand. Otherwise, it is not possible to calculate the test statistic. It may be possible that the difference of our new test statistic is neglecatble in the sample limit, but we don’t have a proof at hand. However, the real difference is use-case setting. If the historic data set with individual patient level data is available, we advise to use the two-sample log rank test. Otherwise, we advise to use our new test methodology which has a similar performance to the two-sample log rank test, but can be used without individual patient level data of the historic cohort.

3. p.4, formula (4): This is not the Greenwood-type variance estimator which is not a problem per se because the existence of hazard rates is assumed, i.e., no ties in the data. However, a relevant fraction or majority of data sets include ties, for which the estimator (4) would result in a biased estimator of the variance. In order to make the paper more relevant and more generally applicable, I suggest to switch to the Greenwood-type estimator. Correspondingly, it would be interesting to see whether the simulation results are affected. (My guess is: probably not much because continuous distributions were used there.) The reason why I am also bringing this up: the historical curves typically also result from data sets that include ties. So, to have a variance estimator that works for the historical data set, one should (also) use the Greenwood-type estimator for it.

Answer:
The reviewer is right, that in Eq. (4) we did not present a Greenwood type variance estimator. However, the premise of the Z-Pi test-statistic was that we do not have to estimate the variance since the factor R can approximate it. Thus, there is no variance estimator for the Nelson-Aalen estimator in the simulations.


Additional major comments:

I. p.5, ll.121-122: I find it surprising that the factor R only depends on the sample sizes. Even under the null hypothesis, I would be surprised. Is it only valid under the condition of equal samplingschemes (and censoring) in both groups? I think some clarification is needed here.

Answer:
Thank you for your comment. The proof is indeed not trivial, but already given in our previous paper under Appendix B. We added this as an additional reference.

II. p.5, ll.128-135 I fail to see the real difference between Z and Z_pi. Is the gist that R is only approximately equal to the reciprocal of sqrt(1+pi)? Does it still matter for big sample sizes? Or does it have to do with my major comment I.?

Answer:
Yes, the difference is that the factor R is only an approximation. From a computational point of view, Z also needs more information to be calculated, whereas Z_pi only needs the classical one sample log rank test statistic and the sample sizes. Furthermore, as stated in corrolary 1 of our previous paper: Both Sigma_OSLR and Sigma converge pointwise almost sure against two variance functions. Thus, the approximation should get better with increasing sample size.


III. p.5, ll.134-135: I don't let this count; in my opinion, knowing the estimated reference curve is almost as good as knowing the historical data set. From the reference curve, one could also retrieve an estimate of sigma_A.

Answer:
We added our take on data reconstruction algorithms in the newly added example, which may also answer the comment by the reviewer.

“Admittedly, reconstruction algorithms deliver solid results when used to reproduce Kaplan Meier point estimators since they are designed to reproduce these as accurately as possible. However using the reconstructed data for estimating hazard ratios or plugging them into a two-sample log-rank test may lead to poor results, as small deviations that are of little relevance for point estimators may add up over the course of the tested time frame \cite{Guyot}. These deviations are not accounted for by the methodology of the two-sample log-rank test and may endanger the correct type I error rate control. This effect is especially prominent when the number of extracted x-y data points from the Kaplan Meier plot image is lower then the number of individual patients data points which need to be reconstructed. In this case the algorithm is under-saturated and has to rely on assumptions.”

Furthermore, we have a counter example at hand, where the two-sample log-rank test finds significant differences between the original and reconstructed data:

We took real world data from approx. 86.000 patients, plotted a Kaplan Meier graph, extracted as much x-y data as the high resolution image allowed and reconstructed the original survival data.

Although only minimal differences can be seen by naked eye, the test result of the two-sample log-rank test is highly significant p=0.006. With a perfect reconstruction algorithm, of course one would have expected a p-value close to 1. This reinforces our view that reconstruction algorithms should not be incorporated into a test methodology without further ado and doing so may inflate the type I error rate unpredictably.

IV. p.6, l.137: what is the motivation/justification for the definition of d at the end p.5, and for the calculation of d at the beginning of p.6?

Answer:
The power of the one-sample log-rank test statistic is driven by the number of observed events, which is denoted by d in the formulas. The upper equation yields a condition on how many events are needed to be observed to gain a certain power against a given significance level. The second equation calculates how many events are expected to be observed in a given trial (depending on sample-size, expected survival and censoring/recruitment scheme). Inserting these two equations into another lets us solve for the unknown design parameters of interest.

Additional remarks and questions:

- p.2, l.56-57: Why does group membership play a role in calendar time but not in study time? This should be explained, and the readability of the sentence could also be improved. Also, another part to correct: "are not simulatenous".

Answer:
Thank you for your remark. We see that the sentence was inducing confusion. In the new version of the manuscript, we rigorously cut out unneeded technical details including this sentence.

- p.3: The definition of J_x seems to be missing.

Answer:
No, it was defined in Line 62, however we omitted J_x entirely since its notation is not needed anymore.


- End of p.3 = end of the section General aspects: This might be a good spot for explaining the need for a test statistic which is different from the classical two-sample test statistic.

Answer:
Thank you for your remark. As suggested, we discussed the need for our new test methodology in more detail.

- p.4, l.117: I guess typical notation for the variance estimator would involve the power 2 (for \hat \sigma_A).

Answer:
We agree with the reviewer, however the notation of \sigma_A was not needed anymore in the new version.

- p.6, l.137: in the definition of omega, is the ratio of the cumulative hazards to be assumed time-constant? This would be a strong restriction in my opinion.

Answer:
Yes, it was assumed for sample size calculation purposes, which is currently the standard implemented in most of the sample-size planning software. Presenting formulas for the non PH alternatives is unfortunately not possible in a compact way. Paradoxically, in our experience, more flexible planning methods are used less frequently by the practical user. The more parameters that have to be defined, the more difficult it is for clinical cooperation partners to supply them. This would also apply to a completely freely selectable alternative survival curve.

- p.6, l.140: What are a_B, f_B, r_B supposed to be? (Knowing this will also facilitate the understanding of (i) and (ii) in l.151.)

Answer:
Thank you for your remark. Indeed, we forgot to define these parameters. We now added the required notation.

- p.7, ll.164-165: there is a index mistake (I believe) in the second display: "c1".

Answer:
Yes, it was by mistake. Thank you for the comment.

- p.7, ll.176-177: "our test with consideration of reference
curve variability, may also be interpreted as a two-sample survival test." Knowing more precisely what is the difference between the proposed novel test and the two-sample test would help understanding this sentence. (Or perhaps reformulate.)

Answer:
Thank you for the comment. We added an example section in which we highlight the differences of the methodologies in more detail.

- p.8, l.206: instead of S_1, it should probably be s on the right-hand side.

Answer:
Yes, correct. Thank you for the remark.

- p.9, ll.249-251: Again, it might not be easy for readers to grasp the essence of this sentence.

Answer:
Thank you for the comment. We think that the confusion stems from the fact that we did not outline the difference of our new test methodology enough, which we now did.

- p.9, ll.257-259: Instead of "interestingly", was this not rather to be expected?

Answer:
Yes, it is an expected result. We rephrased it accordingly.

- p.10, ll.272-273: Again, this sentence might increase confusion. If it has been assumed (implicitly?) that the recruitment and censoring mechanism are similar, this should be made explicit in the paper.

Answer:
Thank you for the comment. We added a more clear statement about this assumption.

- References: Did the authors specify the wrong journal for Ref. 6? I found this paper in Statistics in Biopharmaceutical Research.

Answer:
Thank you for your comment. Yes, we stated the wrong journal by mistake. We corrected it in the new version.
