# Supplementary material for: Two-sample survival tests based on control arm summary statistics
Source: PLoS One. 2024 Jun 14;19(6):e0305434. doi: 10.1371/journal.pone.0305434 (PMC11178201; doi:10.1371/journal.pone.0305434)
Supplement: Attachment — Submitted filename: Response to Reviewers.docx [file pone.0305434.s009.docx]

Answer to editor and reviewer comments

concerning the manuscript ‘Designing one-sample log-rank tests in the absence of historic individual patient data’

now titled ‘Two-sample survival tests based on control arm summary statistics’

(PONE-D-23-29714R1)

We revised the manuscript, following the comments of the reviewers. We aimed to fully implement and consider their very helpful suggestions, as will be detailed below in our answers to the reviewer comments. In particular, we changed the title of the manuscript to acknowledge the two-sample nature of the test. All changes made are highlighted in red color in the ”track changes” version of the manuscript.

**1 Comments of the editor**

(1) I also think that the term "corrected one-sample test" is misleading. In fact the new test it is a two-sample test, which does not require the full individual patient data in one of the 2 samples.

**Answer:**
Thank you for the remark. As suggested, we changed the title of the manuscript to acknowledge the two-sample nature of the test

(2) In the discussion you correctly added the remark that "extreme caution" is required when historical control data are used because this leads to a non-randomized study setting. This point should be acknowledged more thoroughly. I recommend to add that the application of the new test in a non-randomized study setting means that the test can find a significant difference between the 2 compared survival curves, but that it remains unclear whether this difference is due to the treatment or due to confounding. The consequence is the test is suitable for e.g., phase II trials but not for phase III trials as basis for drug approval or HTA decisions.

**Answer:**
Thank you for the comment. We now included this limitation in the discussion.

**Reviewer #1:** 

The revision has strengthened the manuscript. The authors addressed the points raised adequately. Here are some remaining issues which could further strengthen the text.

(1) Upon re-reading the sentence "Here, we propose a corrected one-sample log-rank test that allows to account for the sampling error of the
reference curve without knowledge of the full underlying historical survival time data.", I actually asked myself whether'one-sample' log-rank test is a misnamer for the corrected test procedure. The corrected log-rank test compares a historical with a new data sample, the relevant aspect of the corrected procedure being that, compared to the usual two-sample log-rank test, summary statistics rather than individual patient data are being used for the reference arm. In fact, the very point you make in favour of the corrected procedure is random variability of the inherent assumption for the control arm. Should this indeed be called a one-sample test?

**Answer:**
Thank you for the remark. As suggested, we changed the title of the manuscript manuscript to acknowledge the two-sample nature of the test

(2) Introduction of the example has strengthened the manuscript and serves the technical purpose. The description of the background given previous paradoxical results is also interesting. We don't need to go deep into a subject-matter discussion. However, please consider adding a brief sentence saying that these paradoxical results were not confirmed in your analysis, since the Kaplan Meier curve of the obese is below that of the non-obese.

**Answer:**
Thank you for the comment. As suggested, we added a corresponding sentence to the section.

(3) L. 72f. "We denote by a_B the recruitment period length, with f_B the follow up period length resulting in a trial period length of a_B + f_B for the new intervention trial." Please clarify still further. Is every patient followed up for a period of length only f_B, or is f_B the minimum, a_B + f_B the maximum duration of follow up for the last and first recruited patient, respectively (all patients followed up until calendar time end of trial)? (The latter seems to be the case, according to L. 174ff)

**Answer:**

As suggested, we added two sentences to clarify further. Thank you.

*“We denote by a_B the recruitment period length, with f_B the follow up period length resulting in a trial period length of a_B + f_B for the new intervention trial. In particular, we assume that patients enter the trial between calendar time 0 and a_B, and are then further followed-up until calendar time a_B+f_B, unless they are not censored or experience an event. Therefore the intended follow up of the first patient is a_B+f_B and of the last patient is f_B respectively.”*

(4) L. 307 "Conceptually, the proposed new test also sheds light on a general strategy for lifting existing methodology for single-arm survival trials to a randomized, multi-arm setting" - I find this a somewhat strange statement (can a test shed light on a strategy?). I would rather say that by taking into account the control group variability, your methodology integrates an element into single-armed comparisons with historical control groups that is naturally present in randomized trials, although obviously it still cannot address the potential for selection bias due to the lack of randomization.

**Answer:**
Thank you for the comment. We agree that the sentence was worded strangely. We therefore removed it.

(5) Minor remarks
L. 56 from a group of historical patients, which were treated under standard -> historical patients who (omit German comma)
L. 75 Please introduce the recruitment rate r_B by an equation (presumably n_B/a_B?)
L.96 Φ−1 is the standard normal: Please move to L. 91.5
L. 99 one-sample log-rank test could be used to test H0, if the reference cumulative hazard function Λ_A was known -> test H0 if ... were known (omit German comma)
LL. 132,133: german -> German
LL. 134, 138.5, 146.5: an myocardial -> a myocardial
L. 165: is lower then -> is lower than
L. 173, for all s ≥ s: Please correct
L. 173.5, ... pi. This number of events...: Please reformulate e.g. as '...pi. The number d of events...' (I was confused here because you seemed to describe pi as the number of events).
L. 224 based on the assumption, that: omit German comma
L. 255 "In this case, the breadth of a 95%-confidence interval for an underlying true value of 0.05 is about 0.004". I don't understand this sentence (although I have a guess), please rephrase. True value of what? What confidence interval? None is shown in the tables.
L. 304 f. "Our methodology closes this loophole of artificially reduced sample sizes at the prize of inflated type I error rate." Please rephrase for clarity. A prize has to be paid for the reduced sample size - the sentence could be misread as: for closing the loophole.

**Answer:**
Thank you for the many remarks. We edited the text passages in question.

**Reviewer #2:** 
Thank you for addressing my previous comments.

I only have a minor comment left:
- p. 6, at the end, it currently reads "for all s>=s".

**Answer:**
Thank you for the comment. We corrected the section in question.
